# *FGFR4* Gly388Arg Polymorphism Reveals a Poor Prognosis, Especially in Asian Cancer Patients: A Meta-Analysis

**DOI:** 10.3389/fonc.2021.762528

**Published:** 2021-10-19

**Authors:** Jung Han Kim, Soo Young Jeong, Hyun Joo Jang, Sung Taek Park, Hyeong Su Kim

**Affiliations:** ^1^ Division of Hemato-Oncology, Department of Internal Medicine, Kangnam Sacred-Heart Hospital, Hallym University Medical Center, College of Medicine, Hallym University, Seoul, South Korea; ^2^ Department of Obstetrics and Gynecology, Kangnam Sacred-Heart Hospital, Hallym University Medical Center, College of Medicine, Hallym University, Seoul, South Korea; ^3^ Division of Gastroenterology, Department of Internal Medicine, Dongtan Sacred-Heart Hospital, Hallym University Medical Center, College of Medicine, Hallym University, Hwasung, South Korea

**Keywords:** *FGFR4*, Gly388Arg, polymorphism, prognosis, meta-analysis

## Abstract

The fibroblast growth factor-4 receptor (FGFR4) is a member of receptor tyrosine kinase. The *FGFR4* Gly388Arg polymorphism in the transmembrane domain of the receptor has been shown to increase genetic susceptibility to cancers. However, its prognostic impact in cancer patients still remains controversial. Herein, we performed this meta-analysis to evaluate the clinicopathological and prognostic impacts of the *FGFR4* Gly388Arg polymorphism in patients with cancer. We carried out a computerized extensive search using PubMed, Medline, and Ovid Medline databases up to July 2021. From 44 studies, 11,574 patients were included in the current meta-analysis. Regardless of the genetic models, there was no significant correlation of the *FGFR4* Gly388Arg polymorphism with disease stage 3/4. In the homozygous model (Arg/Arg vs. Gly/Gly), the Arg/Arg genotype tended to show higher rate of lymph node metastasis compared with the Gly/Gly genotype (odds ratio = 1.21, 95% confidence interval (CI): 0.99-1.49, p = 0.06). Compared to patients with the Arg/Gly or Arg/Arg genotype, those with the Gly/Gly genotype had significantly better overall survival (hazard ratios (HR) = 1.19, 95% CI: 1.05-1.35, p *=* 0.006) and disease-free survival (HR = 1.25, 95% CI: 1.03-1.53, p = 0.02). In conclusion, this meta-analysis showed that the *FGFR4* Gly388Arg polymorphism was significantly associated with worse prognosis in cancer patients. Our results suggest that this polymorphism may be a valuable genetic marker to identify patients at higher risk of recurrence or mortality.

## Introduction

The fibroblast growth factor receptors (FGFRs), a subfamily of transmembrane receptor tyrosine kinase (RTK), composed of four related members (FGFR1-4) (1, 2). The activation of FGFR pathway by binding of various ligands to FGFRs triggers several downstream cascades and then activates multiple signal transduction pathways, including the STAT, PI3K/Akt, Ras/Raf/MapK, and phospholipase Cγ (1, 3–5). These signaling pathways regulate a variety of cellular functions such as cell survival, proliferation, migration, differentiation, angiogenesis, and epithelial-mesenchymal transition, and can thereby involve in carcinogenesis (4–6). Actually, numerous studies have demonstrated the aberrant activation of FGFR signaling in carcinogenesis. A recent analysis of 4,853 tumors by the next-generation sequencing has revealed that 7% of cancers carry the FGFR aberrations, including gene amplifications (66%), mutations (26%), and rearrangements (8%) (7). The cancer types most commonly affected were urothelial cancer, breast cancer (BC), endometrial cancer, squamous cell lung cancer, ovarian cancer, carcinoma of unknown primary, glioma and cholangiocarcinoma.

FGFR4 is frequently overexpressed in various types of cancer. It is a highly versatile protein with more than 20 known ligands (8). Specific functions of FGFR4 in carcinogenesis still remain unknown. Whereas Falvella et al. reported downregulation of FGFR4 expression in lung adenocarcinomas (ADC) (9), Sahadevan et al. indicated that FGFR4 expression was upregulated in prostate cancer (PC) (10). The *FGFR4* gene is mapped to chromosome 5 (5q35.1) and is highly polymorphic (11). Single-nucleotide polymorphisms (SNP) are the most common genetic variation, representing 90% of sequence differences with an overall frequency of 1 per 1,000 bases. A common nonsynonymous SNP at codon 388 (rs351855 G>A) located in exon 9, which resulted in the substitution of arginine for glycine (Gly388Arg), was identified in the transmembrane domain of FGFR4 receptor (12). Many researchers have reported that the *FGFR4* rs351855 G>A polymorphism is involved with the development of various types of cancer, including BC (12–14), PC (15, 16), colorectal cancer (CRC) (12, 13, 17), lung cancer (LC) (18–20), hepatocellular carcinoma (HCC) (21, 22), gastric cancer (GC) (23), head and neck squamous cell carcinoma (HNSCC) (24–26), and cervical cancer (27, 28). In the last decade, several meta-analyses were conducted to find that the *FGFR4* Gly388Arg polymorphism was associated with increased risk of some cancers (29–33). In addition, the *FGFR4* Arg388 allele has been linked to advanced stage and more frequent lymph node (LN) metastases than the wild-type homozygote (Gly/Gly) in the cohorts of CRC (12), BC (12, 34), PC (15), and LC (18). It has also been implicated in reducing disease-free survival or overall survival in patients with BC (12, 35), HNSCC (24, 25, 36), GC (37, 38), PC (39), CRC (40), and LC (9, 41). Interestingly, Serra et al. reported that the *FGFR4* Arg388 allele significantly reduced the response to everolimus, an immunosuppressant medication in patients with pancreatic neuroendocrine tumors (panNET) (42). In addition, Thussbas et al. reported that FGFR4 Arg388 was significantly associated with shorter disease-free survival or overall survival among BC patients receiving adjuvant systemic therapy (35). However, other researchers have reported contrasting results in several cancer types (43–46). There have also been many studies that failed to observe any significant contribution of the *FGFR4* Gly388Arg SNP to the clinicopathological parameters or prognosis in patients with cancer (13, 14, 16, 17, 21, 23, 26, 27, 35, 47–65). Therefore, the prognostic impact of the *FGFR4* Gly388Arg polymorphism in cancer patients still remains controversial.

In 2010, Frullanti et al. conducted a meta-analysis of 21 studies to evaluate the role of the *FGFR4* Gly388Arg polymorphism as a prognostic factor in cancers. They found a statistically significant association of the *FGFR4* Arg388 allele and overall survival (hazard ratio (HR) = 1.21, 95% confidence interval (CI) 1.05 – 1.40) and LN involvement (odds ratio (OR) = 1.33, 95% CI 1.01-1.74) (66). Because of a limited number of eligible articles, however, they only included three or less studies in the subgroup analysis according to the primary tumor type. Given the amount of accumulated data thereafter, an updated quantitative synthesis has been deemed worthy. Herein, we performed this meta-analysis to evaluate the clinicopathological and prognostic impacts of the *FGFR4* Gly388Arg polymorphism in patients with cancer.

## Methods

### Publication Search Strategy

This meta-analysis was performed according to the Preferred Reporting Items for Systematic Reviews and Meta-Analyses (PRISMA) guidelines (67). We considered all studies that examined the clincopathological or prognostic value of the *FGFR4* Gly388Arg polymorphism in any types of cancer. We carried out a computerized extensive search using PubMed, Medline, and Ovid Medline databases up to July 2021. The search used the following keywords variably combined: ‘fibroblast growth factor receptor 4 or FGFR4’, ‘polymorphism or SNP’, ‘Gly388Arg or rs351855’ ‘prognosis or survival’ and ‘cancer or carcinoma or tumor.’ The ‘snowball’ method was adopted to identify additional relevant articles and the reference lists of identified articles were hand-searched (68). In case of duplicate publication, the recent paper was selected.

### Eligible Criteria

Studies should meet the following eligible criteria: (i) prospective or retrospective cohort study; (ii) study investigating the association of the *FGFR4* Gly388Arg polymorphism with clinicopathological features (LN metastasis or disease stage) or survival outcomes (disease-free survival or overall survival); (iii) the use of adequate method to assess the *FGFR4* Gly388Arg SNP; (iv) adequate data to estimate OR with 95% confidence interval (CI) for pathological parameters and/or HR with 95% CIs for survival; (v) study published only in peer-reviewed journal; and (vi) article written in English.

### Data Extraction

Two investigators (STP and SYJ), working independently and in parallel, screened the literature and extracted from the eligible articles according to the inclusion criteria. The following data were collected from each article and recorded using the predesigned data-collection form: first author, year of publication, country, ethnicity, inclusion period, sample size, cancer type, genotyping method, genotype counts for the *FGFR4* Gly388Arg polymorphism, LN status, disease stage, survival outcomes (disease-free survival or overall survival), and ORs with 95% CIs for pathological parameters and HRs with their 95% CIs for survival outcomes. When both univariate and multivariate analysis were performed for survival times, the HR with 95% CI from multivariate analysis was selected. Any conflicts were resolved by discussion, with input from the other investigators (JHK and HSK).

### Statistical Analyses

The strength of the association between the *FGFR4* Gly388Arg polymorphism and pathological findings was estimated by the ORs with their 95% CIs in the four genetic models: homozygous [Arg/Arg (AA) vs. Gly/Gly (GG)]; heterozygous (Arg/Gly (AG) vs. GG)); recessive (AA vs. AG+GG); and dominant (AG+AA vs. GG). For the survival analyses, HRs with 95% CIs according to the *FGFR4* Gly388Arg polymorphism status were combined. Statistical values were directly obtained from the original articles. If HRs with their 95% CIs were not reported, the Engauge Digitizer software was utilized to calculate them from the corresponding data and Kaplan-Meier curves. The RevMan version 5.4 software was used to combine the data. The heterogeneity across studies was assessed by the *Q* statistic and *I^2^* inconsistency test. If significant heterogeneity was detected (p  <  0.1 or *I2* > 50%), the random-effects model was selected. Otherwise (p ≥ 0.1 and *I2 ≤*  50%), the fixed-effects model was used. Statistical significance of the pooled HR or OR was determined by *Z* test. The combined OR or HR > 1.0 implies that cancers harboring the *FGFR4* Gly388Arg polymorphism had worse clinicopathological features or survival, respectively.

Publication biases were evaluated by the Begg’s funnel plot (69) and the Egger’s linear regression test (70). All the statistics were two-sided, with p-value < 0.05 considered significant.

## Results

### Results of Search

**Figure 1:**
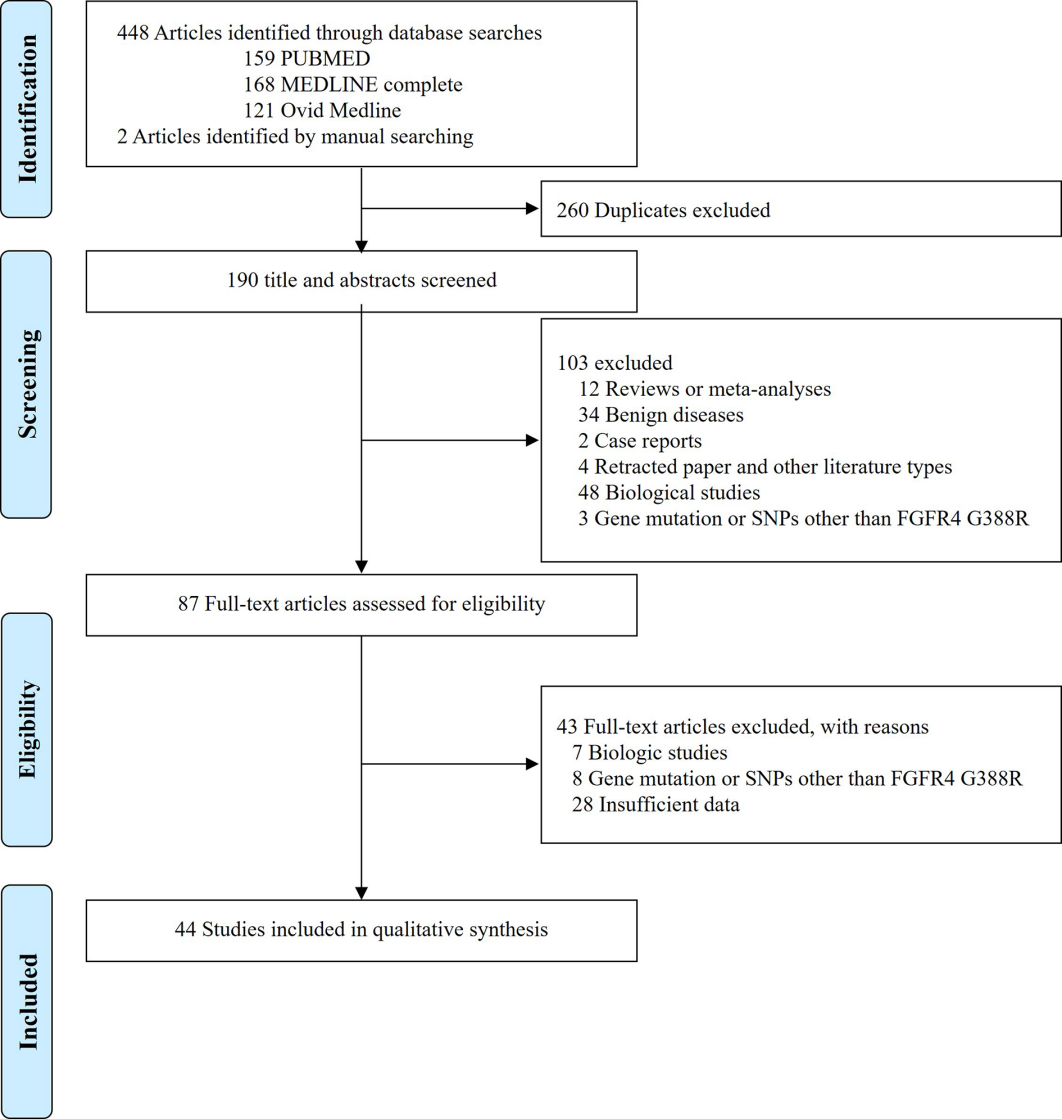
Preferred Reporting Items for Systematic Reviews and Meta-analyses (PRISMA) flow diagram showing the selection process of the included studies.

The flow diagram of search process is shown in **Figure 1**. Except for duplicates, a total of 190 relevant articles were initially retrieved, but 103 of them were excluded after careful screening of the titles and abstracts. Of the remaining 87 potentially eligible studies, 43 which did not meet the eligible criteria were further excluded. Finally, 44 studies were selected for the qualitative synthesis (9, 12–18, 21, 23–27, 34–45, 47–53, 55–65).

### Characteristics of the Included Studies

The main characteristics and clinicopathological findings of the included studies are summarized in **Table 1** and its supplement. In total, data were obtained on 11,574 subjects from the 44 included studies. Studies were more commonly conducted in Western countries (26 studies). Polymerase chain reaction-restriction fragment length polymorphism (PCR-RFLP) method was most frequently used to assess the *FGFR4* Gly388Arg polymorphism status. Most studies used formalin-fixed and paraffin-embedded tissue to extract DNA, except for some utilizing fresh frozen tumor tissue or peripheral blood. The impact of the *FGFR4* Gly388Arg SNP was most often studied in HNSCC (9 studies), followed by LC (8 studies), BC (7 studies), and CRC (6 studies). Other investigated tumor types were GC, PC, HCC, retinoblastoma, sarcoma, melanoma, lymphoma, bladder cancer, ovarian cancer, cervical cancer, and panNET. Twenty-four studies analyzed the pathological or prognostic parameters according to the four genetic models. Seven studies had a small sample size with less than 100 subjects in total (36, 42, 45, 51, 59, 60, 64), and most studies used univariate statistical method to compare survival times, except for seven studies (37, 39, 40, 44, 56, 58, 61).

**Table 1:** The summary of the 44 included studies.

Study (year)	Country	Type of cancer	Genotyping methods	No. of patients(AG/AA vs GG)	Reference
Bange (2002)a	Italy	Breast	PCR-RFLP	84 (43 vs 41)	(12)
Bange (2002)b	Italy	Colon	PCR-RFLP	82 (45 vs 37)	
Morimoto (2003)	Japan	Sarcoma	PCR-RFLP	143 (89 vs 54)	(47)
Becker (2003)	Germany	Breast	PCR-RFLP	246 (141 vs 105)	(34)
Wang (2004)	US	Prostate	RT-RFLP	329 (167 vs 162)	(15)
Jézéquel (2004)	France	Breast	PCR-RFLP	234 (113 vs 121)	(48)
Streit (2004)	Germany	Head and Neck	PCR-RFLP	105 (59 vs 45)	(49)
Spinola (2005)a	Italy	Lung (ADC)	Pyrosequencing	274	(18)
Spinola (2005)b	Italy	Breast	Pyrosequencing	142 (75 vs 67)	(13)
Spinola (2005)c	Italy	Colon	Pyrosequencing	179 (81 vs 98)	
Streit (2006)	Germany	Melenoma	PCR-RFLP	185 (84 vs 101)	(50)
Thussbas (2006)	Germany	Breast	PCR-RFLP	315 (159 vs 156)	(35)
Yang (2006)	US	Bladder	PCR-RFLP	125 (66 vs 59)	(43)
Gordon (2006)	US	Rectum	PCR-RFLP	86 (54 vs 32)	(51)
da Costa Andrade (2007)	Brazil	Head and Neck	PCR-RFLP	75 (31 vs 42)	(36)
Matakidou (2007)	UK	Lung	Illumina Sentrix Bead Arrays	619 (300 vs 319)	(52)
Ma (2008)	Japan	Prostate	PCR-RFLP	492 (329 vs 163)	(16)
Sasaki (2008)	Japan	Lung	RT-PCR	387 (239 vs 148)	(53)
Falvella (2009)a	Italy	Lung (ADC)	Pyrosequencing	541	(9)
Falvella (2009)b	Italy	Lung (SCC)	Pyrosequencing	84	
Falvella (2009)c	Norway	Lung (ADC)	Pyrosequencing	107	
Naidu (2009)	Malaysia	Breast	PCR-RFLP	387 (208 vs 179)	(14)
Tanuma (2010)	Japan	Head and neck (oral SCC)	PCR-SSCP	150 (81 vs 69)	(24)
Ye (2010)	China	Stomach	PCR-RFLP	103 (59 vs 44)	(37)
Azad (2012)	Canada	Head and Neck	Sequenom	528 (247 vs 281)	(55)
Dutra (2012)	Brazil	Head and Neck	PCR-RFLP	122 (56 vs 66)	(56)
Heinzle (2012)	Austria	Colon & rectum	TaqMan assay	182 (106 vs 76)	(17)
Serra (2012)	Canada	Pancreatic NET	PCR-RFLP	71 (36 vs 35)	(42)
Marme (2012)	Germany	Ovary	TaqMan assay	234 (129 vs 105)	(44)
Farnebo (2013)	Sweden	Head and Neck	PCR-RFLP	40 (13 vs 27)	(45)
Shen (2013)	China	Stomach	Sequencing	304 (186 vs 118)	(23)
Gao (2014)	China	Lymphoma	PCR-RFLP	421 (304 vs 117)	(57)
Butkiewicz (2015)	Poland	Lung	PCR-RFLP	348 (195 vs 153)	(58)
Koole (2015)	Netherland	Head and Neck	Sanger sequencing	76 (47 vs 29)	(59)
Sheu (2015)	Taiwan	Liver	TaqMan assay	289 (207 vs 82)	(21)
Chen (2016)	China	Prostate	Sequenom MassArray iPLEX	346 (234 vs 112)	(39)
Cho (2017)	Korea	Colon	Sequencing	273 (181 vs 92)	(40)
Quintanal-Villalonga (2017)	Spain	Lung (SCC)	TaqMan assay	114 (39 vs 75)	(41)
Chou (2017)	Taiwan	Head and Neck (oral SCC)	TaqMan assay	955 (730 vs 225)	(26)
Li (2017)	China	Cervix	PCR-RFLP	162 (127 vs 35)	(27)
Quintanal-Villalonga (2018)	Spain	Lung	TaqMan assay	65 (22 vs 43)	(60)
Wei (2018)	China	Breast	SNaPshot SNP assay	339 (230 vs 109)	(61)
Wimmer (2019)	Germany	Head and Neck	PCR-RFLP	284 (96 vs 188)	(25)
Azuma (2020)	Japan	Liver	TaqMan assay	100 (63 vs 37)	(62)
Li (2020)	Taiwan	Lung	TaqMan assay	277 (201 vs 76)	(63)
Akdeniz Odemis (2020)	Turkey	Retinoblastoma	Sequencing	49 (27 vs 22)	(64)
Ye (2020)	China	Stomach	Sequencing	102 (57 vs 45)	(38)
Shiu (2021)	Taiwan	Colon	TaqMan assay	413 (284 vs 129)	(65)

### Clinicopathological Impact of the *FGFR4* Gly388Arg Polymorphism

**Figure 2:**
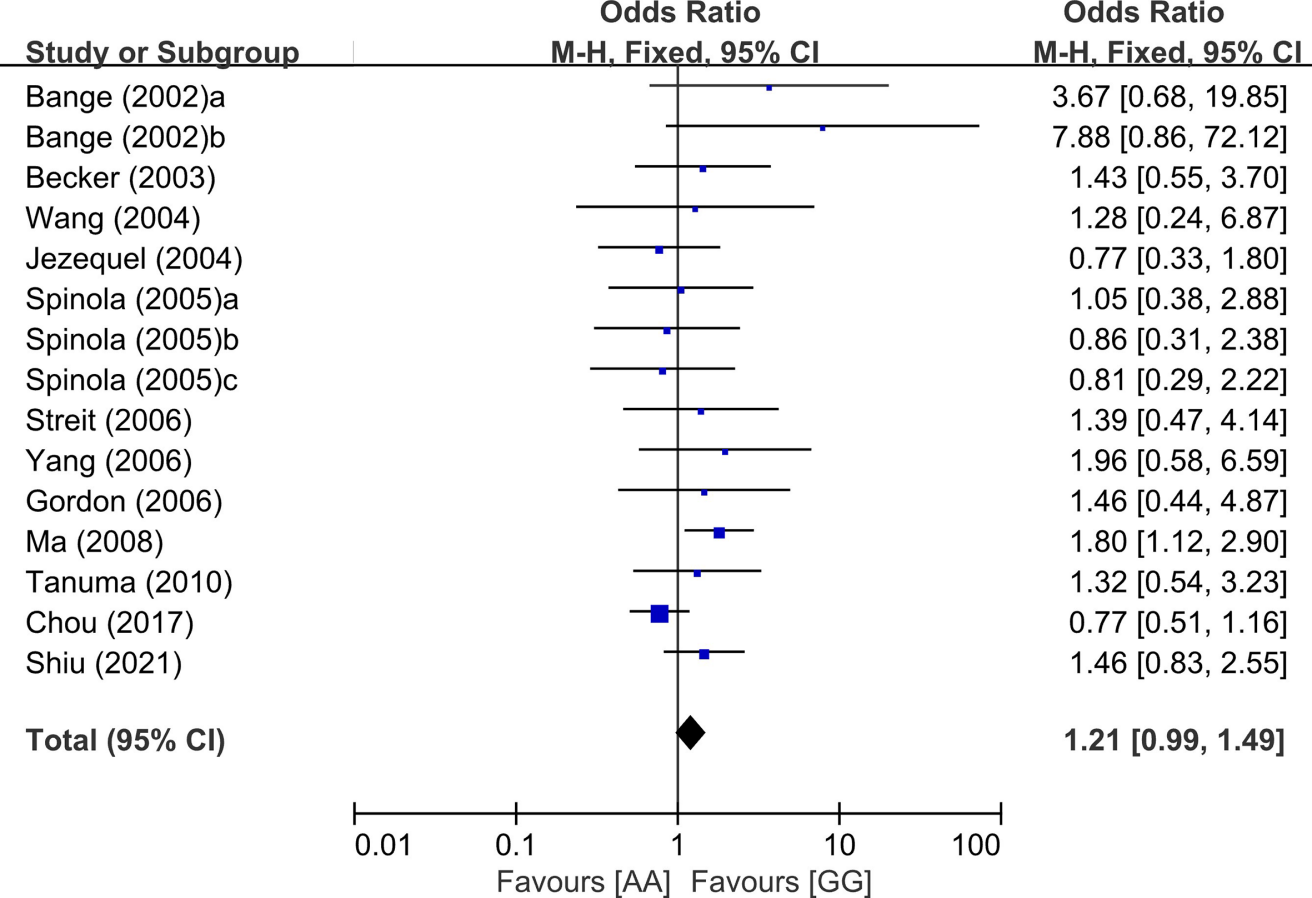
Forest plot for lymph node in the homozygous model (AA vs. GG).

From 24 studies, 6,157 patients were included in the meta-analysis of ORs with 95% CIs for LN metastasis. The odds of LN metastasis at the time of diagnosis were not different between patients with the GG genotype and those with the AG or AA genotype (OR = 1.08, 95% CI: 0.91-1.29, p = 0.39, random-effects, **Table 2**, forest plot not shown). In the homozygous model (amino acid: AA vs. GG), the AA genotype tended to show higher rate of LN metastasis compared with the GG genotype. (OR = 1.21, 95% CI: 0.99-1.49, p = 0.06, fixed-effects) (**Figure 2** and **Table 2**).

**Table 2:** The meta-analyses of clinicopathological and prognostic parameters among the genetic models.

	n	Genetic models	OR or HR (95% CI)			References
			*I^2^*	Fixed-effects	Random-effects	
LN metastases	2725	AG vs GG	54%	1.06 (0.90-1.25) p=0.50	1.13 (0.87-1.47) p=0.36	(12, 13, 15, 16, 18, 24, 26, 34, 43, 48, 50, 51, 65)
	2172	AA vs GG	9%	1.21 (0.99-1.49) p=0.06	1.22 (0.97-1.53) p=0.09	
	3237	AA vs AG+GG	0%	1.02 (0.83-1.25) p=0.85	1.01 (0.82-1.25) p=0.90	
	6157	AG+AA vs GG	53%	1.05 (0.94-1.18) p=0.36	1.08 (0.91-1.29) p=0.39	(12–15, 18, 21, 23–26, 34, 35, 37, 38, 41–44, 48, 51, 53, 56, 63, 65)
Disease stage (3/4)	2325	AG vs GG	31%	0.98 (0.82-1.17) p=0.85	1.02 (0.81-1.29) p=0.86	(13, 16, 18, 26, 27, 43, 49, 50, 64, 65)
	1529	AA vs GG	47%	1.08 (0.86-1.36) p=0.52	1.26 (0.86-1.85) p=0.23	
	2881	AA vs AG+GG	45%	1.13 (0.93-1.38) p=0.21	1.29 (0.93-1.79) p=0.13	
	5585	AG+AA vs GG	42%	1.03 (0.92-1.16) p=0.60	1.05 (0.89-1.25) p=0.56	(9, 13, 14, 16, 17, 21, 23, 26, 27, 37, 41–44, 49, 50, 53, 60, 63–65)
Overall survival	2712	AG vs GG	23%	1.09 (0.97-1.22) p=0.16	1.11 (0.96-1.27) p=0.16	(16, 18, 24, 36, 52, 55, 57–59, 61)
	1869	AA vs GG	76%	1.38 (1.16-1.63) p=0.0002	1.31 (0.91-1.87) p=0.14	
	6607	AG+AA vs GG	63%	1.18 (1.10-1.26) p<0.00001	1.19 (1.05-1.35) p=0.006	(9, 12, 16, 24, 25, 27, 35–38, 40–45, 47, 49, 52, 53, 55–62)

From 21 studies, 5,585 patients were pooled to assess the effect of the *FGFR4* Gly388Arg SNP on disease stage. There was no significant difference of advanced disease (stage 3/4) between patients with the GG genotype and those with the AG or AA genetic type (OR = 1.05, 95% CI: 0.89-1.25, p = 0.56, random-effects, forest plot not shown). The association of the *FGFR4* Gly388Arg SNP with disease stage was not significant in the other genetic models, either (**Table 2**).

### Prognostic Significance of the *FGFR4* Gly388Arg Polymorphism

**Figure 3:**
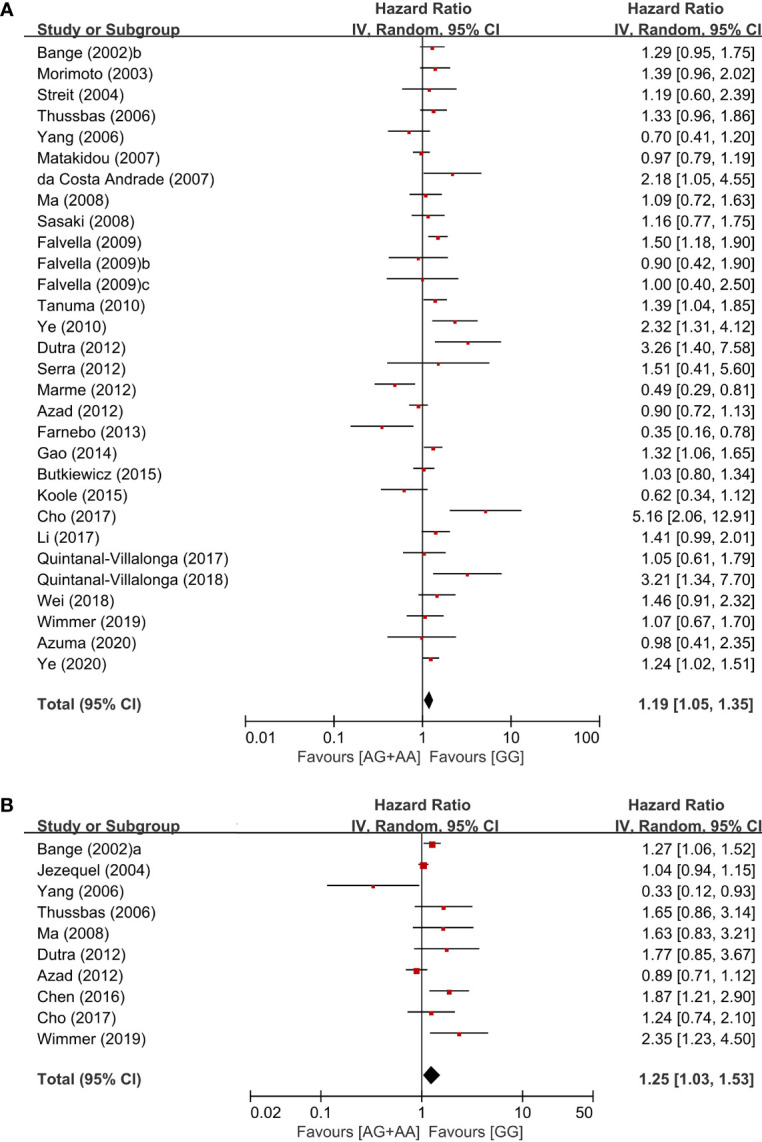
Forest plots for overall survival **(A)** and disease-free survival **(B)** in the dominant model (AG+AA vs. GG).

From 28 studies, 6,607 patients were included in the meta-analysis of HRs for overall survival. Patients with the GG genotype tended to show better overall survival, compared to those with the AG (HR = 1.09, 95% CI: 0.97-1.22, p = 0.16, fixed-effects) or those with the AA genotype (HR = 1.31, 95% CI: 0.91-1.87, p = 0.14, random-effects) (**Table 2**). Compared to patients with the AG or AA genotypes, those with the wild-type homozygote (GG) had significantly longer overall survival (HR = 1.19, 95% CI: 1.05-1.35, p = 0.006, random-effects) (**Figure 3A**).

From 10 studies, 2,803 patients were included in the pooled analysis to evaluate the correlation between the *FGFR4* Gly388Arg SNP and disease-free survival. Patients with the GG genotype showed significantly longer disease-free survival than those with the AG or AA genotype (HR = 1.25, 95% CI: 1.03-1.53, p *=* 0.02, random-effects) (**Figure 3B**).

### Subgroup Analysis According to the Primary Site and Ethnicity

**Figure 4:**
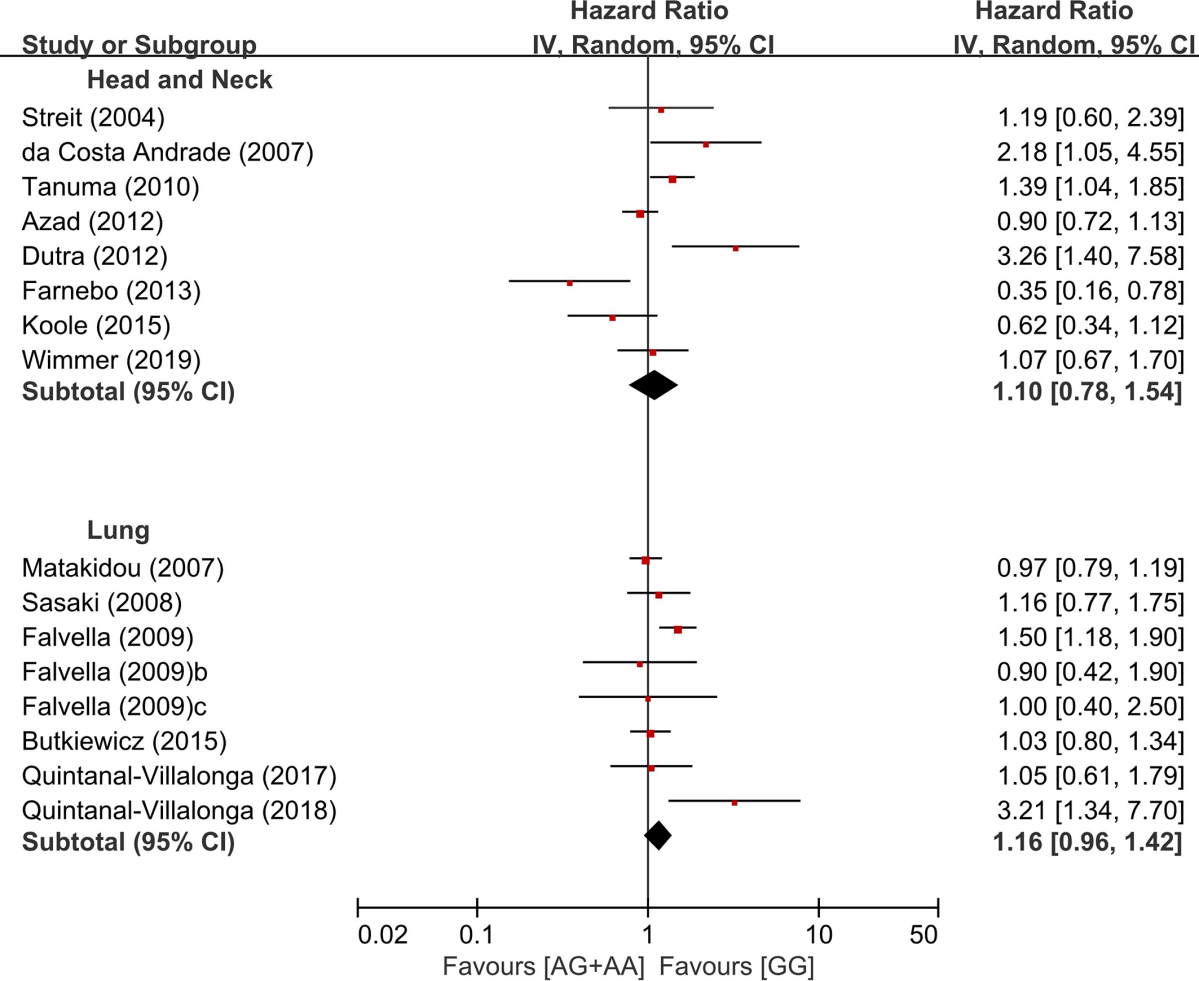
Subgroup analysis according to the primary site.

In the subgroup analysis according to the primary sites, there was no significant overall survival differences between the genotypes of the dominant model (AG+AA vs. GG) in patients with HNSCC (HR = 1.10, 95% CI: 0.78-1.54, p=0.59, random-effects) or LC (HR = 1.16, 95% CI: 0.96-1.42, p = 0.13, random-effects) (**Figure 4**). We did not perform the subgroup analyses for other types of cancer in which only two or less studies were included.

**Figure 5:**
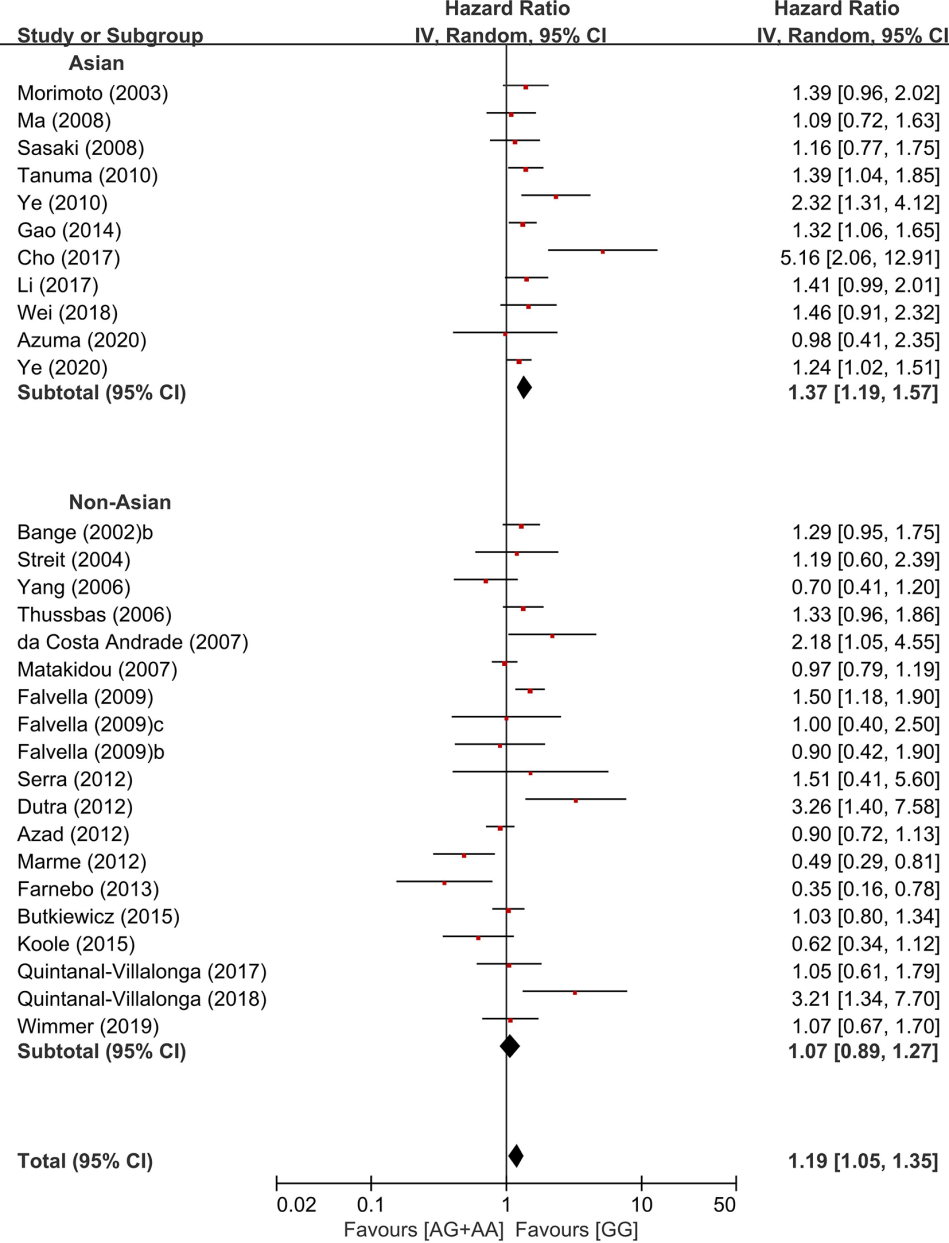
Subgroup analyses according to the ethnicity.

In the stratification by the ethnicity, there was a significant association of the common homozygous genotype (GG) with better overall survival in the Asian population (HR = 1.37, 95% CI: 1.19-1.57, p < 0.00001, random-effects), but not in non-Asians (HR = 1.07, 95% CI: 0.89-1.27, p = 0.47, random-effects) (**Figure 5**).

### Publication Bias

**Figure 6:**
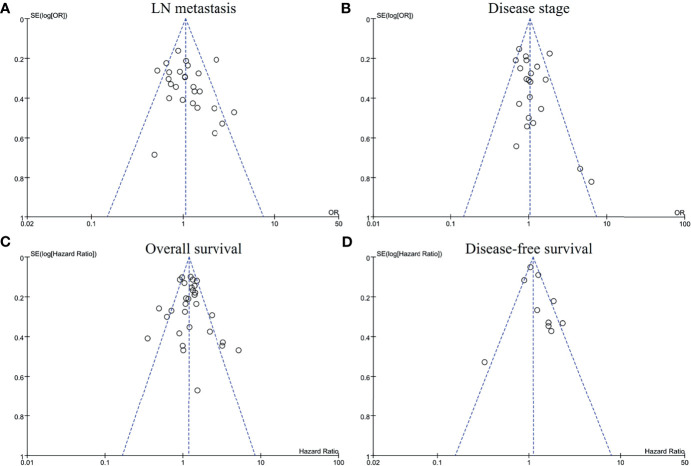
Funnel plots for publication bias regarding lymph node metastasis **(A)**, disease stage **(B)**, overall survival **(C)**, and disease-free survival **(D)**.

The funnel plots of LN metastasis (**Figure 6A**), disease stage (**Figure 6B**), overall survival (**Figure 6C**), and disease-free survival (**Figure 6D**) were graphically symmetric. The Egger’s test indicated no evidence of substantial publication bias for LN metastasis (p = 0.134), stage 3/4 (p = 0.078), overall survival (p = 0.410) and disease-free survival (p = 0.696).

## Discussions

The *FGFR4* Gly388Arg polymorphism results in the replacement of the glycine residue with a charged arginine residue in the transmembrane domain of the receptor, which consequently exposes proximal STAT3 binding site and then enhances STAT3 signal to stimulate cell proliferation (71). Thus, the *FGFR4* Arg388 variants may promote tumorigenesis by enhancing cell motility, invasiveness, and proliferation. The *FGFR4* gene rs351855 G>A polymorphism has been known to confer increased genetic susceptibility to cancers (29–33). Many researchers also examined the relationship between the *FGFR4* gene SNP and its pathological or prognostic roles among diverse cancer types. However, the results were inconsistent. In the current meta-analysis, we evaluated the clinicopathological and prognostic significance of the *FGFR4* Gly388Arg polymorphism in cancers. The study included 11,574 patients with various types of cancer from the 44 eligible articles.

In the current study, the *FGFR4* Gly388Arg SNP failed to show a significant correlation with disease stage 3/4 at the time of diagnosis, regardless of the genetic models. In the homozygous model (AA vs. GG), patients with the AA genotype showed a tendency of higher rate of LN metastases than those with the GG genotype (OR = 1.21, 95% CI: 0.99-1.49, p = 0.06). In terms of survival times, patients with the GG genotype tended to show longer overall survival, compared to those with the AG (HR = 1.09, 95% CI: 0.97-1.22, p = 0.16) or those with the AA genotype (HR = 1.31, 95% CI: 0.91-1.87, p = 0.14). In the dominant model (AG/AA. vs GG), moreover, the Arg388 allele carriers showed a significantly increased hazard of worse overall survival (HR = 1.19, 95% CI: 1.05-1.35, p = 0.006) and disease-free survival (HR = 1.25, 9% CI: 1.03-1.53, p = 0.02). The previous meta-analysis in 2011 by Frullanti et al. reported that there was a significant association between the AA genotype and LN involvement (OR = 1.33, 95% CI: 1.01-1.74, p = 0.04) (66). They also found that the Arg388 allele carriers showed worse prognosis compared to the homozygous carriers of the common Gly388 allele (HR = 1.21, 95% CI: 1.05-1.40, p = 0.01) (66). These results indicate that the *FGFR4* Gly388Arg polymorphism is a potential genetic marker associated with worse prognosis in cancer patients.

The association of the *FGFR4* Arg388 polymorphism with the susceptibility to cancer has mainly been described in PC and BC (15, 16, 29–31, 33). In the meta-analysis of 27 studies comprising 8,682 cases by Xiong et al., interestingly, the *FGFR4* rs351855 G>A polymorphism increased the risk of PC and BC, but decreased the susceptibility of LC (33). This finding indicates that the *FGFR4* Arg388 SNP might has opposite effects on different types of cancer, suggesting that this polymorphism may modify cancer susceptibility in a tissue specific manner. Therefore, the prognostic role of the *FGFR4* Arg388 polymorphism may also be different among different types of cancer. In our subgroup analyses using two most common primary sites, however, there was no significant overall survival difference between the GG and AG/AA genotypes in both HNSCC (HR = 1.10, 95% CI: 0.78-1.54, p=0.65) and LC (HR = 1.16, 95% CI: 0.96-1.42, p = 0.13). In HNSCC, various tumor locations among studies may explain the negative result on the prognostic value of the *FGFR4* Arg388 SNP, since different anatomical locations show different clinical and molecular characteristics (72). In terms of LC, the prognostic impact of the *FGFR4* Arg388 variant may be different among histologic subtypes. Indeed, the prognostic role of this SNP was more frequently observed in patients with ADC (9, 18). In the current study, unfortunately, we could not perform the subgroup analysis according to the tumor location in HNSCC and histologic subtype in LC due to a limited number of relevant articles.

The previous studies have reported that there is significantly different in the prevalence of the *FGFR4* Arg388 allele between Asians (37.2-40.1%) and Caucasians (29.5–30.4%) (29, 33). The higher frequency of the Arg388 allele in Asian populations might lead to a higher statistical power of studies in Asians than in non-Asians. However, the meta-analysis by Xu at al. reported that the association between Arg variant genotype and increase risk of cancer was significant only in Asians, not in Caucacians or Africans (33). In another meta-analysis by Liwei et al., the significant association between Arg variant genotype and susceptibility of PC was observed among European ethic descents, not among African-Americans (31). When we stratified by the ethnicity in the current study, the *FGFR4* Arg388 allele was associated with worse overall survival in Asian population (HR = 1.37, 95% CI: 1.19-1.57, p < 0.00001), but not in non-Asians (HR = 1.07, 95% CI: 0.89-1.27, p = 0.47). These findings suggest that the genetic effect of the *FGFR4* Gly388Arg SNP may be different among the ethnicities.

Beside the *FGFR4* rs351855 G>A polymorphism, there are also other SNPs or mutations of *FGFR4* which may affect the risk of cancer development or prognosis of cancer patients. The recent meta-analysis by Moazeni-Roodi et al. revealed that the *FGFR4* rs1966265 C>T polymorphism significantly reduced the risk of cancer in the recessive model (TT vs CT+CC) and the rs7708357 G>A variant was significantly associated with increased cancer development in the dominant model (AG+AA vs GG) (32). The Y367C *FGFR4* mutation in the extracellular juxtamembrane domain promotes the FGFR4 dimerization on the cell surface and thereby leads to ligand-independent activation of downstream signaling pathways (73). In addition, the mutations in FGFR4 kinase domain such as N535K and V550E cause receptor autophospholyation and then activate the STAT3 signal pathway (73). Some FGFR4 mutations (N535K, V548M and V550L) were reported to be relatively resistant to tyrosine kinase inhibitors (74). However, the clinical and pathological significance of these genetic variations involving *FGFR4* are still needed to be investigated in further studies.

There were some inherent limitations of this meta-analysis. First, there might be a selection bias since we were only able to acquire data from published articles written in English. Second, most studies were performed retrospectively and therefore, may carry the biases of the retrospective design. Third, considerable number of included studies had a relatively small sample size, and most studies utilized univariate statistical method to compare survival outcomes. Forth, because of a paucity of relevant articles, we could not perform the subgroup analyses in other types of cancers than HNSCC and LC. Finally, there was a substantial heterogeneity in the pooled outcomes, which might weaken reliability of the meta-analysis although the random-effects model was adopted.

In conclusion, this meta-analysis elucidated that *FGFR4* Gly388Arg polymorphism was associated with worse prognosis in cancer patients. Our results suggest that this SNP may be a valuable genetic marker to identify patients at higher risk of recurrence or mortality. Considering the limitations of the current study, however, large prospective researches with genotyping of the whole *FGFR4* locus are warranted to reveal the clinicopathological and prognostic roles of the *FGFR4* Gly388Arg SNP among various types of cancer, histology, and ethnicity.

## Data Availability Statement

The original contributions presented in the study are included in the article/**Supplementary Material**. Further inquiries can be directed to the corresponding authors.

## Author Contributions

JHK and HSK conceived and designed the study. STP and SYJ searched the literatures and extracted the data. HSK and STP carried out the statistical analyses and data interpretation. JHK and HJJ wrote the manuscript. All authors contributed to the article and approved the submitted version.

## Funding

This work was supported by the Bio & Medical Technology Development Program of the National Research Foundation (NRF) and funded by the Korean government, Ministry of Science and ICT (MSIT) (NRF- 2020R1G1A1005483).

## Conflict of Interest

The authors declare that the research was conducted in the absence of any commercial or financial relationships that could be construed as a potential conflict of interest.

## Publisher’s Note

All claims expressed in this article are solely those of the authors and do not necessarily represent those of their affiliated organizations, or those of the publisher, the editors and the reviewers. Any product that may be evaluated in this article, or claim that may be made by its manufacturer, is not guaranteed or endorsed by the publisher.
