# Supplementary material for: FGFR4 Gly388Arg Polymorphism Reveals a Poor Prognosis, Especially in Asian Cancer Patients: A Meta-Analysis
Source: Front Oncol. 2021 Oct 19;11:762528. doi: 10.3389/fonc.2021.762528 (PMC8560792; doi:10.3389/fonc.2021.762528)
Supplement: Supplementary file 1 [file Table_1.docx]

**Supplement Table 1** The summary of the 44 included studies.

| **Study (year)** | **Country** | **Type of cancer** | **Genotyping methods** | **No. of patients**  **(AG/AA vs GG)** | **LN mets**  **(AG/AA vs GG)** | **Stage 3 & 4**  **(AG/AA vs GG)** | **HR for OS (95% CI)**  **p-value (m or u)** | **HR for DFS (95% CI)**  **p-value (m or u)** | **Reference** |
| --- | --- | --- | --- | --- | --- | --- | --- | --- | --- |
| Bange (2002)a | Italy | Breast | PCR-RFLP | 84 (43 vs 41) | 29/43 vs 20/41 | NA | NA | 1.27 (1.06-1.52)  p=0.009 (u) | (12) |
| Bange (2002)b | Italy | Colon | PCR-RFLP | 82 (45 vs 37) | 33/45 vs 16/37 | NA | 1.29 (0.952-1.747)  p=0.1 (u) | NA |  |
| Morimoto (2003) | Japan | Sarcoma | PCR-RFLP | 143 (89 vs 54) | NA | NA | 1.39 (0.956-2.022)  p=0.085 (u) | NA | (47) |
| Becker (2003) | Germany | Breast | PCR-RFLP | 246 (141 vs 105) | 36/141 vs 26/105 | NA | NA | NA | (34) |
| Wang (2004) | US | Prostate | RT-RFLP | 329 (167 vs 162) | 16/158 vs 5/125 | NA | NA | NA | (15) |
| Jézéquel (2004) | France | Breast | PCR-RFLP | 234 (113 vs 121) | 66/113 vs 73/121 | NA | NA | 1.04 (0.94-1.15)  p=0.46 (u) | (48) |
| Streit (2004) | Germany | Head and Neck | PCR-RFLP | 105 (59 vs 45) | NA | 46/59 vs 32/45 | 1.19 (0.60-2.39)  p=0.615 (u) | NA | (49) |
| Spinola (2005)a | Italy | Lung (ADC) | Pyrosequencing | 274 | 66/119 vs 28/139 | NA | 1.6 (1.1 – 2.3)*  p=0.08 (u) | NA | (18) |
| Spinola (2005)b | Italy | Breast | Pyrosequencing | 142 (75 vs 67) | 34/74 vs 28/64 | NA | NA | NA | (13) |
| Spinola (2005)c | Italy | Colon | Pyrosequencing | 179 (81 vs 98) | 42/81 vs59/97 | 53/80 vs 64/98 | NA | NA |  |
| Streit (2006) | Germany | Melenoma | PCR-RFLP | 185 (84 vs 101) | NA | 31/84 vs 19/101 | NA | NA | (50) |
| Thussbas (2006) | Germany | Breast | PCR-RFLP | 315 (159 vs 156) | 57/159 vs 52/156 | NA | 1.332 (0.956-1.855)  p=0.09 (u) | 1.646 (0.863-3.138)  p=0.13 (u) | (35) |
| Yang (2006) | US | Bladder | PCR-RFLP | 125 (66 vs 59) | 29/66 vs 22/59 | 56/66 vs 50/59 | 0.704 (0.414-1.197)  p=0.198 (u) | 0.33 (0.117-0.930)  p=0.036 (u) | (43) |
| Gordon (2006) | US | Rectum | PCR-RFLP | 86 (54 vs 32) | 30/54 vs 15/32 | NA | NA | NA | (51) |
| da Costa Andrade (2007) | Brazil | Head and Neck | PCR-RFLP | 75 (31 vs 42) | NA | NA | 2.184 (1.048-4.550)  p=0.037 (u) | NA | (36) |
| Matakidou (2007) | UK | Lung | Illumina Sentrix Bead Arrays | 619 (300 vs 319) | NA | NA | 0.97 (0.79-1.19)  p=0.78 (u) | NA | (52) |
| Ma (2008) | Japan | Prostate | PCR-RFLP | 492 (329 vs 163) | NA | 116/329 vs 51/163 | 1.09 (0.72-1.63)  p=0.69 (u) | 1.63 (0.83-3.21)  p=0.16 (u) | (16) |
| Sasaki (2008) | Japan | Lung | RT-PCR | 387 (239 vs 148) | 64/239 vs 53/148 | 92/239 vs 71/148 | 1.157 (0.766-1.746)  p=0.489 (u) | NA | (53) |
| Falvella (2009)a | Italy | Lung (ADC) | Pyrosequencing | 541 | NA | 142/252 vs 114/278 | 1.5 (1.1-1.9)  p=0.0068 (u) | NA | (9) |
| Falvella (2009)b | Italy | Lung (SCC) | Pyrosequencing | 84 | NA | NA | 0.9 (0.42-1.90) (u) | NA |  |
| Falvella (2009)c | Norway | Lung (ADC) | Pyrosequencing | 107 | NA | NA | 1.0 (0.40-2.50) (u) | NA |  |
| Naidu (2009) | Malaysia | Breast | PCR-RFLP | 387 (208 vs 179) | 122/206 vs 69/179 | 54/195 vs 40/173 | NA | NA | (14) |
| Tanuma (2010) | Japan | Head and neck  (oral SCC) | PCR-SSCP | 150 (81 vs 69) | 26/81 vs 25/69 | NA | 1.39 (1.04-1.85)  p=0.03 (u) | NA | (24) |
| Ye (2010) | China | Stomach | PCR-RFLP | 103 (59 vs 44) | 36/59 vs 27/44 | 38/59 vs 31/44 | 2.324 (1.054-4.125)  p=0.037 (m) | NA | (37) |
| Azad (2012) | Canada | Head and Neck | Sequenom | 528 (247 vs 281) | NA | NA | 0.90 (0.72-1.13)  p=0.37 (u) | 0.89 (0.71-1.12)  p=0.33 (u) | (55) |
| Dutra (2012) | Brazil | Head and Neck | PCR-RFLP | 122 (56 vs 66) | 32/56 vs 31/66 | NA | 3.26 (1.40-7.58)  p=0.006 (m) | 1.77 (0.85-3.67)  p=0.124 (m) | (56) |
| Heinzle (2012) | Austria | Colon & rectum | TaqMan assay | 182 (106 vs 76) | NA | 52/106 vs 28/76 | NA | NA | (17) |
| Serra (2012) | Canada | Pancreatic NET | PCR-RFLP | 71 (36 vs 35) | 11/35 vs 6/35 | 10/35 vs 2/34 | 1.507 (0.406-5.595)  p=0.54 (u) | NA | (42) |
| Marme (2012) | Germany | Ovary | TaqMan assay | 234 (129 vs 105) | 70/103 vs 61/82 | 122/129 vs 101/105 | 0.49 (0.29-0.81)  p=0.006 (m) | NA | (44) |
| Farnebo (2013) | Sweden | Head and Neck | PCR-RFLP | 40 (13 vs 27) | NA | NA | 0.348 (0.156-0.777)  p=0.01 (u) | NA | (45) |
| Shen (2013) | China | Stomach | Sequencing | 304 (186 vs 118) | 131/186 vs 91/118 | NA | NA | NA | (23) |
| Gao (2014) | China | Lymphoma | PCR-RFLP | 421 (304 vs 117) | NA | NA | 1.32 (1.06-1.65)  p=0.015 (u) | NA | (57) |
| Butkiewicz (2015) | Poland | Lung | PCR-RFLP | 348 (195 vs 153) | NA | NA | 1.03 (0.80-1.34)  p=0.809 (m) | NA | (58) |
| Koole (2015) | Netherland | Head and Neck | Sanger sequencing | 76 (47 vs 29) | NA | NA | 0.62 (0.34-1.12)  p=0.114 (u) | NA | (59) |
| Sheu (2015) | Taiwan | Liver | TaqMan assay | 289 (207 vs 82) | 5/207 vs 4/82 | 71/207 vs 27/82 | NA | NA | (21) |
| Chen (2016) | China | Prostate | Sequenom MassArray iPLEX | 346 (234 vs 112) | NA | NA | NA | 1.873 (1.209-2.901)  p=0.005 (m) | (39) |
| Cho (2017) | Korea | Colon | Sequencing | 273 (181 vs 92) | NA | NA | 5.161 (2.062-12.916)  p<0.001 (m) | 1.243 (0.735-2.102)  p=0.417 (u) | (40) |
| Quintanal-Villalonga (2017) | Spain | Lung  (SCC) | TaqMan assay | 114 (39 vs 75) | 15/34 vs 26/68 | 18/39 vs 34/75 | 1.047 (0.611-1.794)  p=0.867 (u) | NA | (41) |
| Chou (2017) | Taiwan | Head and Neck  (oral SCC) | TaqMan assay | 955 (730 vs 225) | 230/730 vs 77/225 | 361/730 vs 127/225 | NA | NA | (26) |
| Li (2017) | China | Cervix | PCR-RFLP | 162 (127 vs 35) | NA | 108/127 vs 30/35 | 1.41 (0.99-2.01)  P=0.057 (u) | NA | (27) |
| Quintanal-Villalonga (2018) | Spain | Lung | TaqMan assay | 65 (22 vs 43) | NA | 11/22 vs 20/43 | 3.21 (1.34-7.90)  p=0.002 (u) |  | (60) |
| Wei (2018) | China | Breast | SNaPshot SNP assay | 339 (230 vs 109) | NA | NA | 1.456 (0.915-2.317)  p=0.113 (m) | NA | (61) |
| Wimmer (2019) | Germany | Head and Neck | PCR-RFLP | 284 (96 vs 188) | 70/96 vs 122/188 | NA | 1.07 (0.67-1.70)  p=0.7754 (u) | 2.35 (1.23-4.50)  p=0.01 (u) | (25) |
| Azuma (2020) | Japan | Liver | TaqMan assay | 100 (63 vs 37) | NA | NA | 0.98 (0.41-2.35)  p=0.963 (u) |  | (62) |
| Li (2020) | Taiwan | Lung | TaqMan assay | 277 (201 vs 76) | 142/201 vs 57/76 | NA | NA | NA | (63) |
| Akdeniz Odemis (2020) | Turkey | Retinoblastoma | Sequencing | 49 (27 vs 22) | NA | 24/27 vs 14/22 | NA | NA | (64) |
| Ye (2020) | China | Stomach | Sequencing | 102 (57 vs 45) | 28/57 vs 26/45 | NA | 1.24 (1.02-1.51)  p=0.033 (u) | NA | (38) |
| Shiu (2021) | Taiwan | Colon | TaqMan assay | 413 (284 vs 129) | 139/284 vs 61/129 | 143/284 vs 67/129 | NA | NA | (65) |
